# Tailoring a response to youth binge drinking in an Aboriginal Australian community: a grounded theory study

**DOI:** 10.1186/1471-2458-13-726

**Published:** 2013-08-07

**Authors:** Janya McCalman, Komla Tsey, Roxanne Bainbridge, Anthony Shakeshaft, Michele Singleton, Christopher Doran

**Affiliations:** 1 The Cairns Institute, James Cook University, PO Box 6811, Cairns, QLD 4870, Australia; 2 University of New South Wales, Sydney, Australia; 3 Gindaja Treatment and Healing Centre, Yarrabah, Australia; 4 University of Newcastle, New South Wales, Australia

**Keywords:** Indigenous population, Binge drinking, Adolescent, Community-based participatory research, Research design

## Abstract

**Background:**

While Aboriginal Australian health providers prioritise identification of local community health needs and strategies, they do not always have the opportunity to access or interpret evidence-based literature to inform health improvement innovations. Research partnerships are therefore important when designing or modifying Aboriginal Australian health improvement initiatives and their evaluation. However, there are few models that outline the pragmatic steps by which research partners negotiate to develop, implement and evaluate community-based initiatives. The objective of this paper is to provide a theoretical model of the tailoring of health improvement initiatives by Aboriginal community-based service providers and partner university researchers. It draws from the case of the Beat da Binge community-initiated youth binge drinking harm reduction project in Yarrabah.

**Methods:**

A theoretical model was developed using the constructivist grounded theory methods of concurrent sampling, data collection and analysis. Data was obtained from the recordings of reflective Community-Based Participatory Research (CBPR) processes with Aboriginal community partners and young people, and university researchers. CBPR data was supplemented with interviews with theoretically sampled project participants. The transcripts of CBPR recordings and interviews were imported into NVIVO and coded to identify categories and theoretical constructs. The identified categories were then developed into higher order concepts and the relationships between concepts identified until the central purpose of those involved in the project and the core process that facilitated that purpose were identified.

**Results:**

The tailored alcohol harm reduction project resulted in clarification of the underlying local determinants of binge drinking, and a shift in the project design from a social marketing awareness campaign (based on short-term events) to a more robust advocacy for youth mentoring into education, employment and training. The community-based process undertaken by the research partnership to tailor the design, implementation and evaluation of the project was theorised as a model incorporating four overlapping stages of *negotiating knowledges and meanings* to *tailor a community response*.

**Conclusions:**

The theoretical model can be applied in spaces where local Aboriginal and scientific knowledges meet to support the tailored design, implementation and evaluation of other health improvement projects, particularly those that originate from Aboriginal communities themselves.

## Background

Aboriginal Australians are well aware of the health and social harms resulting from alcohol misuse. In response, they have initiated many interventions [1]. However a paucity of evidence about what works to ameliorate Aboriginal alcohol-related harms has meant that decisions regarding the choice and delivery of interventions have been generally founded either on the evidence from mainstream populations or on local informal or qualitative assessments [1,2].

Local assessments from community consultations are fundamental to identifying needs and tailoring feasible and acceptable Aboriginal alcohol harm reduction initiatives, and need to be at the forefront of ethical and cost-effective research endeavours [3]. Nevertheless it is not always possible for community service providers to independently design evidence-based programs or services or robust evaluation plans. Community-based organisations themselves, for example, do not have ready access to the intervention or implementation literatures to ensure that their programs are informed by the best available evidence [4]. Similarly, they do not typically have the expertise to design practical and methodologically robust evaluation plans and measures. Research partnerships are therefore important for bringing together local Aboriginal knowledge and scientific knowledge when designing or modifying alcohol harm-reduction initiatives and their evaluation. However, despite the role that research partnerships can play in informing Aboriginal health improvement approaches [5-7], there are few models that outline the pragmatic steps by which research partners negotiate to develop, implement and evaluate tailored community-based initiatives.

The objective of this paper is to elucidate a theoretical model for tailoring community-based Aboriginal health initiatives. The theory is grounded in a whole-of-community alcohol harm-reduction project targeting young people in the north Queensland Aboriginal community of Yarrabah: the Beat da Binge project. Beat da Binge aimed to ameliorate the harms from binge drinking, or drinking to intoxication by heavy consumption of alcohol over a short period of time [8]. This theoretical paper complements a second paper which provides the pre/post evaluation results of a community survey of Yarrabah young people’s awareness of, and behaviours related to binge drinking (Jainullabudeen, A., Jacups, S., Shakeshaft, A., Doran, C, Tsey, K. "Beat da Binge" : Impact of an anti-binge-drinking intervention in an Indigenous community, in preparation). Both papers report on the work of a collaborative research partnership involving the Yarrabah alcohol rehabilitation service, Gindaja Treatment and Healing Indigenous Corporation (hereafter Gindaja) and seven other Yarrabah community organisations with researchers from James Cook University (JCU), Hunter Medical Research Institute (HMRI) at the University of Newcastle and the National Drug and Alcohol Research Centre (NDARC) at the University of New South Wales.

## Methods

### Study design

This research partnership used a strengths-based approach implemented through Community Based Participatory Research (CBPR) as the qualitative component of a mixed methods approach. CBPR provided a framework for mutual interchange between researchers and community partners aiming to make the research relevant to the needs of the community [9]. As an iterative approach, CBPR is consistent with the cyclical processes of sampling, data collection and analysis of grounded theory methods. Constructivist grounded theory methods were therefore used to theorise the key Beat da Binge design, implementation and evaluation processes discussed in the CBPR sessions. Further, constructivist grounded theory is considered appropriate to the task of conducting exploratory research in situations such as Aboriginal youth binge drinking, where there has been little prior research. The methods are also well suited to encompassing the particular ethics of care and responsibility that are requisite in Indigenous research methodologies [10,11]. The protocol for the research project was approved by JCU Ethics Committee (H 3532).

### Setting

Originally a church mission, Yarrabah is a discrete north Queensland community of 2409 residents; 97 per cent of whom are Aboriginal [12]. The median age of Aboriginal residents is 21 years and half are aged 25 years or less [12]. Based on income, job status, occupation, personal qualifications, service availability and housing conditions, the community is Australia’s most disadvantaged local government area [13]. The Beat da Binge project was prompted by the closure of the work-for-the-dole Community Development Employment Program (CDEP) in Yarrabah in July 2009, as in all non-remote Aboriginal communities across Australia. For young people, the impact of the CDEP job losses on engagement and wellbeing was exacerbated by a reduction in youth activities since 2006 due to the reallocation of the youth centre and community hall to other community priorities.

Community leaders became concerned that the cessation of CDEP could escalate binge drinking among young people and exacerbate violence, crime, accidents, suicide and family stress [1]. Although the community had been a restricted alcohol area^a^ since 2004, residents had ready access to alcohol from the nearby southern suburbs of Cairns and illegal unlicensed alcohol vendors (sly groggers) within the community. Binge drinking was prevalent at parties on dole days and for coming of age celebrations for 18 and 21 year olds. Led by Gindaja, eight community organisations^b^ assembled to plan a whole-of-community approach to harm reduction. They were successful in their funding submission to the National Binge Drinking Strategy to implement Beat da Binge.

### Intervention

Beat da Binge was a two-year project that commenced in April 2010. It was designed to prevent harm from binge drinking for Yarrabah young people through seeking to alleviate boredom and a sense of futility and anger among youth and other community members; and promote self-empowerment, achievement and pride (Funding Application, 2009). The whole-of-community social marketing approach incorporated “prevention and awareness strategies to help overcome social and emotional wellbeing problems in the Yarrabah community incurred by the misuse of alcohol” (Funding Application, 2009). A project reference committee comprising a representative from each of the eight community partners planned to provide a year-round program of two major events and twelve minor activities. At each event, consistent harm reduction messages would be provided to educate Yarrabah’s young people about alcohol misuse, high risk times, and appropriate responses to alcohol. Events, including Foundation Day (the commemoration of the founding of the mission in 1893) and NAIDOC week (National Aborigines and Islanders Day Observance Committee which celebrates the history, culture and achievements of Aboriginal and Torres Strait Islander peoples), as well as agency-driven music, sporting and cultural events were implemented to the clientele of local organisations. Young people aged 12–24 years were the primary target, but activities were flexibly designed to be inclusive of all community members. In the first year, these events attracted 1880 participants, with an average age of 16 (Report to funding body, 2011). Additionally, low cost cultural resources such as T-shirts and leaflets were produced.

### Participants and data collection

Parallel to this community-based process, and based on long-established and mutually beneficial partnerships between community organisations and researchers, researchers from JCU, HMRI and NDARC were invited to evaluate Beat da Binge and to join the project steering committee. Researchers initiated reflective quarterly CBPR meetings with the project steering committee and young Yarrabah people who were actively involved in the project as research assistants, members of a youth reference sub-group and Youth Council leaders. The steering committee members and young people were asked to respond to the findings of a literature review, undertaken by researchers, about the harms caused by binge drinking among Aboriginal young people, relevant responses to binge drinking and theoretical frameworks for harm reduction responses. As well, the steering committee reflected on the results of a survey of young Yarrabah people. The survey had been designed by the Beat da Binge project officer in consultation with young people; refined and augmented in partnership with researchers to ensure survey items were reliable, valid and comprehensive; and reviewed and implemented by Yarrabah young people who were trained and remunerated as research assistants. Reflections included explorations of the extent to which any changes observed from pre- to post-intervention might be attributable to the BDB activities. CBPR data were collected from the 16 project steering committee members, 18 (8 male/10 female) young people, and three researchers who participated in these processes.

**Figure 1:**
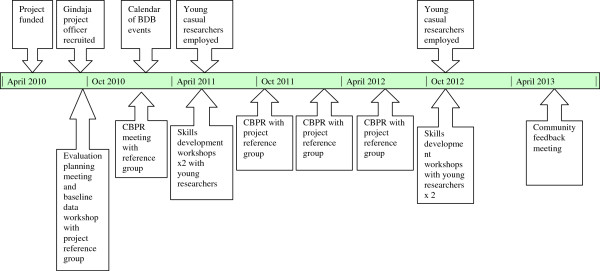
Timeline of intervention and data collection events.

Theoretical constructs identified through early analysis of the CBPR data were further explored through targeted in-depth interviews with three of the community partners, three young people and two researchers. Interviewees were selected based on the grounded theory method of theoretical sampling; that is, sampling of those who were likely to provide divergent views on emergent theoretical issues. As well, project documents were referenced. Interviewees were asked towards the end of the project to reflect on what had worked well about the project design and implementation processes, what had not worked well, and what they considered were the key lessons from the approach. The key events relating to the intervention and data collection processes are provided in Figure 1.

### Analysis

The transcripts of CBPR recordings and interviews were imported into NVIVO and coded to identify recurrent themes and theoretical constructs [10]. The identified themes were then repeatedly categorised into higher order concepts and the relationships between constructs identified. The modelling process continued until the theorist was satisfied that the constructed model explained the great majority of the data, and she had identified the central concern of those involved in the project and the basic process that facilitated that concern [10]. The model, developed from the grounded data, was corroborated by project steering committee members. The constructed model and supporting qualitative data are presented below.

## Results

**Figure 2:**
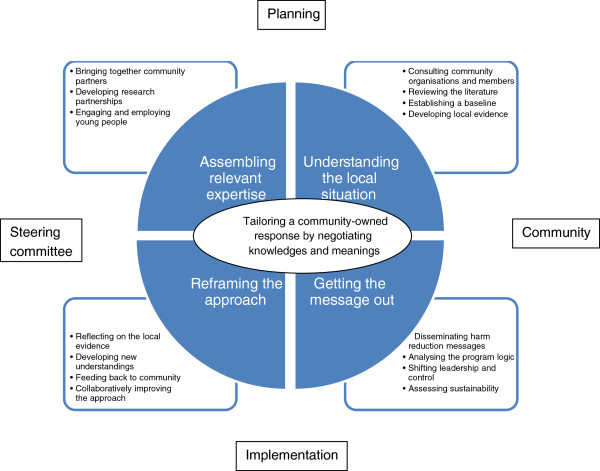
**Proposed theoretical model: *tailoring a community response *to alcohol harm by *negotiating knowledges and meanings.***

The purpose of research partners, and core concern of the constructed theoretical model, was *tailoring a community response* to alcohol harm. A community partner described Beat da Binge as: “tailored from the local situation. And like those stakeholders that were involved, we got them involved in the initial writing of the application so they were aware of what was going on …”. The core process by which the community response was tailored was *negotiating knowledges and meanings*. This referred to the efforts of the steering committee to align local Aboriginal knowledges and broader scientific evidence to find the most effective way to reduce alcohol harm among young people in Yarrabah. *Negotiating knowledges and meanings* was experiential and required four inter-related and transformative overlapping stages (to be discussed in detail in the subsequent sections). As a result of these stages, a community leader reflected: “it got better as we went along I think.” Figure 2 depicts the model of *tailoring a community response*.

### Assembling expertise

The first stage of the model involved *assembling* the *expertise* of three groups: Yarrabah community organisations, researchers and local young people. Pro-actively, Yarrabah organisations led by Gindaja had identified the funding source, developed the funding proposal and formed a steering group, independently of external expertise. Community partners perceived that the whole-of-community approach would reduce young people’s binge drinking as well as benefit their organisations through financial sponsorship for events and activities. A community leader considered this whole of community approach to be effective:

“Having the whole-of-community focus, you make it the whole community’s responsibility, not just one organisation … I think was a better approach.”

The same community leader also reflected that community involvement was likely to increase the acceptability of the approach to government funders:

“Using the whole-of-community approach, we know government likes that kind of stuff so we thought well, we’ll go that way. There was interest from other stakeholders in the community, so that was good.”

The capacity of the eight organisations to engage in the partnership approach varied according to their perceived level of influence in the decision making processes and the degree of benefit they perceived for their organisation from their involvement. This was unsurprising given their other commitments [14]. Nevertheless, five of the eight organisations remained routinely involved in organising events and activities during the first year.

Once the project had been funded, research partners were invited to help evaluate Beat da Binge. A research partner recalled:

“We were not part of it to start with. We had actually gone to Gindaja to consult them about a different project …. During that process it became clear to them that there was an opportunity for them to make use of our expertise. So they said, look we have a more pressing project, that being, Beat da Binge”.

Researchers first conducted a literature review, identifying evidence for the effectiveness of positioning young people at the centre of health initiatives that targeted their demographic. Six months into Beat da Binge, young people in Yarrabah were invited to help with the project implementation and evaluation. Two young people joined the steering committee, but their initial engagement was erratic. The minutes of a steering committee meeting in October 2010 stated: “We need young people at the table - we need young people’s views about why these problems arise”. A researcher recalled: “we sat, a group of us adults, service providers and researchers, sat around a table just trying to work out whether it’s possible at all, and how to engage young people in the Beat da Binge”.

Two new strategies were implemented to improve the engagement with young people. First, young Yarrabah people were trained in interview techniques, and paid to opportunistically survey other young people, independently of Beat da Binge events, in the park, at the school, and with friends and family members. Four were employed to conduct pre-intervention surveys (March 2011) and seven post-intervention surveys (May 2012). They provided a core group of young people who contributed to steering committee meetings for the duration of their short-term employment. Second, a Yarrabah Youth Council, established in October 2010, began to liaise closely with the Beat da Binge steering committee. The president of the Youth Council stated their willingness to: “fill in the potholes and help out where we can”.

### Understanding the local situation

The second stage of the model comprised the development of a clear *understanding* of *the local situation* by the three expert groups (Yarrabah organisations, researchers and young people). Community partners had an experiential understanding of the nature and extent of binge drinking among young people. One community leader reflected: “we live here and we see what’s going on”. They perceived that binge drinking had been exacerbated by restrictions on the supply of alcohol^i^ as well as the closure of the Yarrabah CDEP in July 2009. A community leader recalled: “everyone had gone back onto Centrelink [government welfare] payments, and a lot of drinking and that was going on in the community, because CDEP did keep them in an active role two days a week where they wouldn’t be drinking.” As a result of CDEP cessation, a community leader observed:

“We’re seeing our youth starting their drinking careers on cheap cask wine and its bad binge drinking. … Whereas before some of the restrictions come in, it was a lot of the pre-mix stuff. That way, it’s portioned properly; you know their drinking amounts.… I think if we don’t start dealing with it soon, in ten years, we’ll have a young generation of alcoholics, you know, on cask wine.”

Researchers searched the literature to identify strategies most likely to reduce harms associated with binge drinking, but found only eleven publications that described or evaluated alcohol harm reduction projects for Aboriginal young people [15-25]. All eleven publications were qualitative evaluations or descriptions of pilot projects, which included school education, music, sporting, community-driven and peer education approaches, and youth-specific substance misuse services. No studies had demonstrated statistically significant changes in alcohol consumption, school attendance, youth apprehensions or other indicators. More locally, no evaluation of the effects of the alcohol supply restrictions or the closure of CDEP on patterns of alcohol consumption in Yarrabah was found.

This lack of evidence provided a rationale to more rigorously determine the nature and extent of young people’s binge drinking and the strategies most likely to reduce harms. Consistent with the participatory intent of the project, community partners, young people and researchers provided strategic feedback on the development of a survey instrument. All eight community organisations routinely involved in the steering committee agreed that the survey could be distributed at their events.

### Getting the message out

The third stage of the model related to the project implementation and comprised the dissemination of alcohol harm reduction messages about high risk times and appropriate responses to alcohol, thus *getting the message out*. All Yarrabah community organisations were invited to submit proposals to implement alcohol-free community activities and a calendar of events was drawn up. In return for event sponsorship by Beat da Binge, community organisations were expected to: 1) distribute promotional Beat da Binge messages; 2) complete an events feedback form comprising participant data; and 3) administer the survey as widely as possible at their events. A competition resulted in an approved logo design for all promotional materials. By the end of the first year, nine activities had been delivered. They included the Seahawks rugby league club opening performance of the season, the All Black’s football carnival, two ‘dive-in’ movie nights, a barbecue lunch, carols by candlelight, a Foundation Day event, and taekwondo and boxing training. But the program logic analysis suggested that the events were not consistent with the project aim of reducing binge drinking. Based on the literature, researchers advised that it was unlikely that the social marketing approach of *Beat da Binge* would have sufficient leverage to change either the binge drinking behaviour of Aboriginal young people or the environments in which drinking occurred.

In the second year, the steering committee decided to shift the locus of control for organising events to young people. This shift was fuelled by an increasing awareness of the need to place young people at the centre of the project, as well as a lack of compliance by some community partners in completing the written funding application and activity reporting forms, administering the survey or delivering Beat da Binge messages. A steering committee member observed: “Some people saw it as a bucket of money and didn’t comply with *Beat da Binge* requirements. To me, it was money that didn’t promote the aims of *Beat da Binge* as well as it could”.

Subsequent youth-organised activities demonstrated that young people were learning for the first time about concepts such as the definition of a ‘standard drink’ and about how they could reduce risks from drinking alcohol. One young person, for example, commented: “standard drink, I didn’t know there’s standard drink”. A young woman commented that while some young “people drink to get drunk, a lot of us drink just to have a few.” Another young man concurred that although there was a culture of grog parties in Yarrabah, his preference was to: “drink in moderation, not in desperation”. A steering committee member concurred with the young people’s comments, stating: “youth night was awesome – it showed that you don’t have to get drunk to enjoy yourself. There was no disharmony. If they get drunk, then they get jealous and want to fight”.

Reflecting on the achievements of *Beat da Binge* eighteen months into the project, a community partner perceived the project to have been successful in *getting* harm reduction *messages out*. She reflected:

“I think we’ve got about six months left of the *Beat da Binge*, we’ve got the messages out there as much as possible. We talk about it all the time, and a lot of promotional gear going out so kids are talking about it, they’re taking the stuff home so they’re talking about it at home. But I think there’s other solutions for this, and it’s going to take a lot more to fix it up than just a two year promotional program.”

With the exception of boxing and taekwondo training, *Beat da Binge* comprised a series of one-off events, the short-term impact of which was described by the project coordinator as “disheartening”. The perceptions and experiences of young people were further examined to determine more effective and sustained solutions to youth binge drinking.

### Reframing the approach

The fourth stage of the model comprised *reframing the approach*. In October 2011, the results of the baseline survey were presented at a steering committee meeting incorporating five representatives from the Youth Council. The survey results confirmed community partners’ perceptions: young people reported consuming alcohol because they were bored and disengaged, lacking employment, training or other life opportunities. Asked through CBPR to reflect on the meaning of boredom in the context of why young people were binge drinking, a Youth Council member stated: “they don’t have any direction in life; no meaning or purpose”. Other comments included: “lack of goal”; “nothing to aim or fight for and no support for that”; “no job and training”; “nothing to do all day”; and “nothing going on”. Boredom therefore referred to a deeper lack of purpose, engagement or meaning in life for young people, and not to a lack of activity or entertainment. Binge drinking provided a way of creating social connectedness with peers and relief from a cycle of disengagement and lack of hope for the future.

In addition to one-off events, it appeared that attention to the socio-economic and cultural factors associated with young people’s disengagement from education, employment and training in Yarrabah could be usefully explored. The longer-term challenge for the *Beat da Binge* project partners therefore became the consideration of how they might better support young people to foster meaningful lives by overcoming the structural barriers to education, employment or training. One young person commented: “A lot of people do training in Yarrabah but they don’t do anything afterwards. There should be more job opportunities.” A Youth Council member reflected:

“If we want to break the cycle, we could engage the good kids and the borderline kids – if we could get them over and drag the other kids with them….There’s a low chance of getting a job in Yarrabah but we need to build them up to go out of the community because that’s the only real place that economic opportunities exist. Many are afraid to go out – they need to know how to handle money, how to budget, how to speak on their rights etc. It’s just all about empowering”.

Similarly, another Youth Council member suggested:

“…youth … positive rather than still with their head down, still unsure. And that they have that awareness and expectations of future engagement”.

The reframing of the approach towards engagement into meaningful education, employment and training was confirmed at a community meeting held in March 2013 to feed back the research results. The meeting was attended by representatives of six of the eight Beat da Binge project steering committee organisations and other Yarrabah community members. Community service providers reiterated a need for long term strategies that prompted Yarrabah young people’s life purpose and a frustration at and mistrust of unsustainable one-off funded initiatives. Stimulated by a presentation of the Back Track mentoring model [26], participants demonstrated continued interest in pursuing youth mentoring and other empowerment strategies and discussed the feasibility of reframing the approach to explore options for youth mentoring and support models that do not necessarily depend on government funding.

### Limitations

The model provides one theoretical construction, among many other possible interpretations, of how Aboriginal community responses to health issues can be tailored. In particular, the strengths-based approach meant that the primary focus was on data about what worked in designing and implementing the project rather than what didn’t work, or why the program was not sustained. However, the credibility of the findings was enhanced by the participatory nature of the research process, familiarity with the setting and topic, systematic comparisons between the data and categories, and logical links between the gathered data and analysis.

The model was based on the participatory process of designing and evaluating only one alcohol harm reduction program. The nature of the program may mean that the model is directly applicable only to alcohol harm reduction programs or only to Aboriginal Australian programs. The similarities with experiences of the research team in designing and implementing health projects in Aboriginal Australian and other settings [7,27,28], however, suggest that the key elements of the theoretical model could provide a useful blue print for the design and implementation of community-based services and programs more generally. However, this would need to be determined on a case-by-case basis.

## Discussion and conclusions

Aboriginal scholars and organisations, and research institutions have advocated the involvement of research partnerships in developing, implementing and evaluating community-based health improvement approaches. But the process of negotiating decisions regarding the tailoring and implementation of feasible and acceptable Aboriginal health initiatives has been problematic for many research partnerships. Developing authentic research relationships is complicated, and the time restrictions of short-term funded projects and differing expectations and priorities compound difficulties.

In Yarrabah, the process of tailoring a community response to binge drinking led Gindaja to win a National Drug and Alcohol Award for excellence in services for young people in June 2013 [29]. More broadly, the theoretical model of *tailoring a community-owned response* by *negotiating knowledges and meanings* provides a framework for systematically strengthening the evidence base for how research partnerships can tailor effective health improvement approaches by integrating local Aboriginal knowledges and assessments with the scientific evidence. The model prompts project initiators to engage those targeted by a project as well as community stakeholders and researchers in its design and implementation; to analyse local understandings of the health issue and integrate these understandings with the scientific evidence; to ensure that the project activities are consistent with and likely to achieve the project aim; and to be open to reframing the approach if this proves not to be the case. Table 1, derived from the model, outlines the overlapping stages and sub-processes that can be considered and applied by research partnerships to tailor community responses in other situations. It is critical that such Aboriginal voices and perspectives are included in genuine dialogue about practice and policy evidence that affects their interests [30].

**Table 1:** **The stages of *negotiating knowledges and meanings *to *tailor a community response***

**Stage**	**Sub-processes**
Assembling expertise	Initiating the project by community organisation/s.
	Developing partnerships with researchers.
	Engaging and employing community members who are the target of the project.
Understanding the local situation	Consulting with community organisations and members.
	Reviewing the relevant intervention and implementation literature.
	Establishing a baseline.
	Developing local evidence.
Getting the message out	Disseminating health promotion messages through social marketing.
	Analysing the program logic to determine whether the project aim and strategies are consistent.
	Shifting the locus of control for project strategies to community members who are the target of the project.
	Assessing the sustainability of the approach.
Reframing the approach	Reflecting on the local evidence and project experience.
	Developing new understandings of the determinants.
	Feeding back project evaluation results to broader community stakeholders.
	Collaboratively improving the approach to address revised understandings of the determinants and consider issues of sustainability.

## Endnotes

^a^Alcohol restrictions limit alcohol carried per person to one carton of 30 cans of light or mid-strength beer OR one 750 ml bottle of unfortified wine (DATSIP 2010). It is an offence to drink in a public place. Penalties apply to people breaching these restrictions (DATSIP 2010).

^b^The Aboriginal Shire Council, community-controlled health service, justice group, women’s shelter and resource centre, rugby league football and sports club, state school, Police and Citizens Youth Club and church.

## Competing interests

The authors declare that they have no competing interests.

## Authors’ contributions

JMC took a primary role in the paper’s conception and design, acquisition of data, and analysis and interpretation of data using grounded theory methods. KT advised on the paper’s conception, was involved in data acquisition revised the manuscript and gave final approval for publication. RB contributed to acquisition of data, advised on interpretation of data using grounded theory methods, and revised the manuscript for cultural appropriateness and intellectual content. AS critically revised the manuscript for alcohol harm reduction concepts and intellectual rigour. MS contributed to acquisition of data and contributed to Gindaja’s approval of the manuscript for publication. CD critically revised the manuscript for intellectual rigour. All authors read and approved the final manuscript.

## Pre-publication history

The pre-publication history for this paper can be accessed here:

http://www.biomedcentral.com/1471-2458/13/726/prepub

## References

[B1] Wilson M The harmful use of alcohol amongst Indigenous Australians 2010 Accessed from http://www.healthinfonet.ecu.edu.au/alcoholuse_review, August 2013

[B2] Paul C Being sorry is not enough. The sorry state of the evidence base for improving the health of Indigenous populations Am J Prev Med 2010 38 5 566 568 10.1016/j.amepre.2010.02.001 20409504

[B3] National Health and Medical Research Council Values and Ethics: Guidelines for Ethical Conduct in Aboriginal and Torres Strait Islander Health Research 2003 Canberra: Commonwealth of Australia 24

[B4] McCalman J Applying what works: a systematic search of the transfer and implementation of promising indigenous Australian health services and programs BMC Public Health 2012 12 600 10.1186/1471-2458-12-600 PMC3490811 22856688

[B5] O’Neil J Reader J Leader A Changing the relations of surveillance: the development of a discourse of resistance in Aboriginal epidemiology Human organization 1998 57 2 230 237

[B6] Mayo K The research dance: university and community research collaborations at Yarrabah, North Queensland, Australia Health Soc Care Community 2009 17 133 140 10.1111/j.1365-2524.2008.00805.x 18800983

[B7] Bainbridge R A partnership approach to transitioning policy change in Aboriginal Australian communities J Aust Indigenous Issues 2013 15 1 55 75

[B8] Renaud S Diet and stroke J Nutr Health Aging 2001 5 3 167 172 11458287

[B9] Bainbridge R Coming to an ethics of research practice in a remote Aboriginal Australian palliative care setting: a grounded theory study Rural and Remote Health In press

[B10] Glaser BG Strauss AL The discovery of grounded theory; strategies for qualitative research 1967 Chicago: Aldine Pub. Co 271

[B11] Bainbridge R McCalman J Whiteside M Being, knowing and doing: a phronetic approach to constructing grounded theory with Indigenous partners Qual Health Res 2012 23 2 275 288 23208201 10.1177/1049732312467853

[B12] Australian Bureau of Statistics Census quick stats 2013 Canberra, Australia: Australian Bureau of Statistics

[B13] Australian Bureau of Statistics (ABS) Census of Population and Housing 2008 Australia: Socio-Economic Indexes for Areas (SEIFA) Data only, 2006

[B14] McCalman J Taking control of health: Gurriny’s story of organisational change Third Sector Review 2010 16 1 29 49

[B15] Bentley M Evaluation of the peer education component of the Young Nungas Yarning Together program 2008 Adelaide: South Australian Community Research Unit 21

[B16] Holyoake Institute for Drug and Alcohol Addiction Resolutions and Midlands Education Aboriginal Office, An evaluation of the music therapy intervention “Drumbeat” with alienated youth in the wheatbelt of Western Australia NSW: Family Action Centre 2010 Newcastle: University of Newcastle

[B17] Sheehan N Alcohol education in an indigenous community school in Queensland 1995 Education, Prevention and Policy: Australia. Drugs

[B18] Gray D Sputore Walker Evaluation of an Aboriginal health promotion program: a case study from Karalundi 1998

[B19] Milne Josif Lynn Raypirri:evaluation of the Northern Territory Bush tour project April-June 1993 1993 Canberra: DHHLGC Public Affairs Branch

[B20] Stathis SL Developing an integrated substance use and mental health service in the specialised setting of a youth detention centre Drug Alcohol Rev 2006 25 2 149 155 10.1080/09595230500537613 16627304

[B21] Lee K Evaluation of a community-driven preventive youth initiative in Arnhem land, Northern Territory, Australia Drug Alcohol Rev 2008 27 1 75 82 10.1080/09595230701711124 18034384

[B22] Murray D Holloway C Koori youth alcohol and drug healing service - The first two years Drug Alcohol Rev 2009 28 1 A45

[B23] Dinan Thompson M Sellwood J Carless F A Kickstart to life: Australian football league as a medium for promoting lifeskills in Cape York Indigenous communities Aust J Indigenous Educ 2008 37 152 164

[B24] Gugan Gulwan Youth Aboriginal Corporation Drug and alcohol program (Gugan Gulwan Youth Aboriginal Corporation) 2012 17/09/2012]; Available from: http://www.gugan-gulwan.com.au/services/4-drug-and-alcohol

[B25] Indigenous Wellbeing Centre Youth, alcohol, drug treatment and support program (YAD) 2012 Available from: http://www.healthinfonet.ecu.edu.au/key-resources/programs-projects?pid=1106

[B26] Youth Action and Policy Association New South Wales Backtrack 2011

[B27] Tsey K A micro analysis of a participatory action research process with a rural Aboriginal men’s health group Aust J Prim Health 2004 10 64 71 10.1071/PY04009 12472608

[B28] McCalman J Bringing us back to our origin: adapting and transferring an Indigenous Australian values-based leadership capacity building course for community development in Papua New Guinea 2011 Community Development: Journal of the Community Development Society

[B29] QNADA New Congratulations to Gindaja and QLD Police Service on Drug Action week 2013 Accessed from: http://www.qnada.org.au/news/666/congratulations-to-gindaja-and-QLD-police-service-on-drug-action-week-awards

[B30] Maddison S Evidence and contestation in the Indigenous policy domain: voice, ideology and institutional inequality Aust J Public Adm 2012 71 3 269 277 10.1111/j.1467-8500.2012.00775.x

